# Evolution of multimodal intraoperative neuromonitoring in pediatric spinal deformity surgery over 39 years: are we better?

**DOI:** 10.1007/s43390-026-01453-4

**Published:** 2026-06-11

**Authors:** Gabrielle A. Rogie, Hilton C. Braithwaite, Chris Bozorgmehr, Leah Rakers, Brian A. Kelly, Scott J. Luhmann

**Affiliations:** 1 Department of Orthopedic Surgery, Washington University in St. Louis School of Medicine, St. Louis, MO USA; 2 Department of Electrophysiology, Barnes-Jewish Hospital, St. Louis, MO USA

**Keywords:** Intraoperative neuromonitoring, Spinal cord monitoring, Pediatric spinal deformity, Neuromonitoring alerts, Evolution of IONM

## Abstract

**Purpose:**

To describe the incidence and characteristics of multimodal intraoperative neuromonitoring (IONM) in pediatric spinal deformity surgery between two eras (1985–2008 vs. 2008–2024).

**Methods:**

A review was conducted of 3316 consecutive pediatric spinal deformity surgeries (2008–2024) at a single institution. Neuromonitoring modalities included somatosensory-evoked potentials (SSEP), transcranial electrical motor-evoked potentials (TCeMEP), and descending neurogenic-evoked potentials (DNEP). Due to inherent cohort heterogeneity and evolving surgical standards over four decades, a descriptive analysis was prioritized to characterize shifts in monitoring performance and case complexity.

**Results:**

IONM alerts were more frequent in 2008–2024 at 4.1% (1 in 24) versus 2.2% (1 in 46). Highest alert rates were in kyphosis/kyphoscoliosis (14.8% vs. 9.3%) and revision procedures (9.5% vs. 3.0%). Most alerts occurred during correction/instrumentation (62.2% vs. 47%). Permanent neurologic deficits were more common in the modern cohort: 16 patients (0.48%, 1 in 207) versus 6 (0.17%, 1 in 573). False negatives occurred at similar frequencies (0.15% vs. 0.2%) but were more consequential, with 80% resulting in permanent deficits versus 14% previously. While lower in comparison to the historic era, IONM was associated with a disparity between alert frequent and observed permanent neurologic deficit rates with alerts occurring in 4.1% of cases and permanent deficits in 0.48% (historic era: 2.2 and 0.17%, respectively). However, this coincided with an increase in case complexity as evidenced by higher prevalence of three-column osteotomies and use of traction.

**Conclusion:**

Multimodal IONM remains a critical adjunct in pediatric spinal deformity surgery, with alert events occurring in 4.1% of cases and an observed permanent neurologic deficit rate of 0.48%. False negatives, particularly nerve root related, remain a key limitation. Observed differences between eras likely reflect evolving case complexity, surgical techniques, and monitoring practices rather than changes in intrinsic monitoring performance.

**Supplementary Information:**

The online version contains supplementary material available at https://doi.org/10.1007/s43390-026-01453-4.

## Introduction

Multimodal intraoperative neurophysiologic monitoring (IONM) has become the standard of care in spinal deformity surgery, aiming to reduce the risk of iatrogenic neurologic deficit during corrective procedures [1, 2]. A 2010 retrospective study by Thuet et al. reviewed 23 years of pediatric spinal deformity surgical cases from 1985 to 2008 at a single institution. The study reported the efficacy of multimodality IONM in identifying and reducing permanent neurologic deficits across a wide range of spinal pathologies in pediatric patients [2].

Since 2008, shifts have occurred in modalities of IONM due to advancements in technology, knowledge, surgical technique and anesthesia, as well as studies furthering understanding of IONM modalities [3, 4]. As the complexity of pediatric spinal surgery has increased, so has the need for standardized protocols when IONM changes occur. Current literature emphasizes the importance of systematic responses to intraoperative alerts, particularly in high-risk pediatric coronal spinal deformity and three-column posterior spinal osteotomies [5]. Furthermore, the development of consensus-based best practice guidelines has provided a framework for managing these events in high-risk surgery, though their application across evolving surgical eras remains a subject of ongoing investigation [6, 7]. To our knowledge, no studies have reassessed the impact of these advancements in multimodal IONM across large pediatric cohorts. To address this need, our study expands upon the original dataset by analyzing pediatric spinal deformity surgeries performed between 2008 and 2024 at the same institution. By evaluating neurologic complication rates, alert events, and diagnostic category-specific outcomes between the two eras, we aim to assess how the use of multimodal IONM has evolved over nearly four decades of continuous practice.

The purpose of this study is to describe the incidence and predictive performance of intraoperative neurophysiologic monitoring in pediatric spinal surgery between two periods: 1985–2008 (historic) and 2008–2024 (modern). Our specific aims are (1) to describe the rate of permanent and transient neurologic deficits in both time periods, (2) to characterize changes in monitoring events, and (3) to evaluate whether specific diagnostic subgroups continue to benefit equally from IONM over time. We hypothesize that the performance metrics of multimodal IONM have remained stable over time; however, shifts in monitoring modalities and surgical techniques (such as the increased use of VCRs and pedicle screw constructs) have altered the clinical presentation and frequency of intraoperative alerts between the two eras.

## Methods

This study is a retrospective descriptive cohort analysis conducted at a single tertiary pediatric institution. Data from a modern cohort (2008–2024) were evaluated alongside a previously published historical cohort (1985–2008). Due to substantial differences between eras in patient characteristics, surgical techniques, and perioperative management, formal comparative statistical inference was not emphasized, and findings are presented descriptively.

An institutional neuromonitoring database was queried to identify all patients 18 years of age or under who underwent spinal deformity surgery at one of our three hospitals from 2008 to 2024. Neuromonitoring and demographic data were collected and retrospectively analyzed. Surgeries were performed by one of seven fellowship-trained orthopedic surgeons at a single institution. All diagnostic categories with neuromonitoring reports for the spinal deformity surgery were included. The query identified only pediatric spine cases—excluding neurosurgical spine cases including as Chiari decompressions, tethered cord releases, and central nervous system tumor resections. Subgroup analyses were performed by diagnostic category, including adolescent idiopathic scoliosis, neuromuscular scoliosis, congenital deformity, kyphosis/kyphoscoliosis, revision surgery, and other less common etiologies, to assess differences in alert frequency and neurologic outcomes.

Primary multimodal IONM consisted of somatosensory-evoked potentials (SSEPs), descending neurogenic-evoked potentials (DNEPs), or transcranial motor-evoked potentials (TCeMEPs), and triggered electromyography (EMG) pedicle screw testing. Prior to the institution of TCeMEPs at our center, DNEPs were used, and the transition between modalities is reported in this analysis. Further details about the use of each modality at the time of surgery, warning criteria, and anesthetic regimen are detailed in the Online Appendix.

Intraoperative monitoring outcomes were classified into four categories:

- *True positive* (TP) Data met warning criteria, and either intervention resolved the alert, or patient awoke with deficit on exam
- *True negative* (TN) Stable evoked data with no postoperative deficit
- *False positive* (FP) Data met warning criteria, no documented intervention occurred, and no postoperative deficit occurred
- *False negative* (FN) No data change, but postoperative deficit occurred

**Fig. 1:**
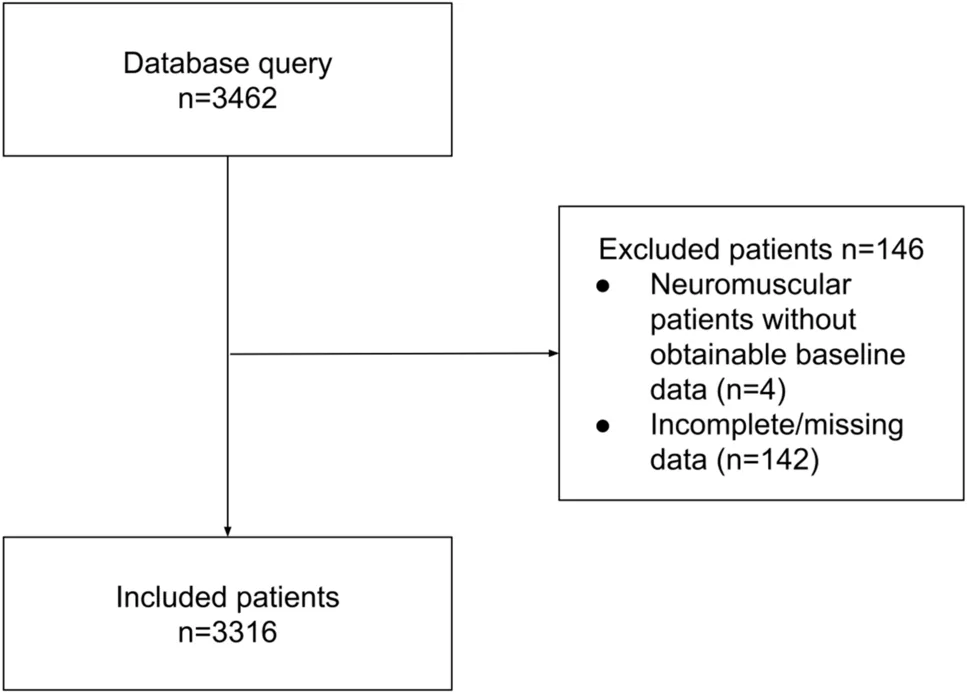
Study inclusions/exclusions

Database query identified 3462 patients. Four neuromuscular patients in which alerts occurred were excluded due to baseline function it could not be determined if these were in fact true or false positives. One hundred forty-two additional patients were excluded for incomplete or missing intraoperative monitoring reports. A total of 3316 consecutive pediatric patients who underwent spinal deformity surgery between 2008 and 2024 and were included. Figure 1 demonstrates the inclusions and exclusions. Statistical analysis for baseline demographics included two-proportion *Z* tests and Fischer’s exact with *p* < 0.05 being statistically significant.

## Results

In the modern cohort there were 3316 pediatric patients (66% female; *n* = 2174) whose mean age was 13 years (range, 2 to 18 years), which was statistically similar to the historic cohort with 64% female and mean age of 13 years (range, 13 months to 18 years). There were 135 alerts (4.1%) in the modern cohort, and 74 (2.2%) in the historic cohort. In the modern cohort there were two fewer surgeons and 120 more patients than in the 1985–2008 study. The most common diagnoses in the modern cohort included idiopathic scoliosis (1835, 55.3%), neuromuscular scoliosis (480, 14.5%), and kyphosis/kyphoscoliosis (270, 8.1%). The most common diagnoses for the historic cohort were also idiopathic scoliosis (1618, 47%) and neuromuscular scoliosis (626, 18.2%). However, the third most common diagnosis in this cohort was revision (329, 9.6%), Table 1. There were significant differences in diagnostic distribution in the cohorts for the numbers of idiopathic (*p* < 0.0001), neuromuscular (*p* < 0.0001), revision (*p* < 0.0001), kyphosis/kyphoscoliosis (*p* = 0.0139), spondylolisthesis (*p* = 0.001), and degenerative (*p* = 0.001) patients. The highest percentage of alerts in the modern cohort were in patients with kyphosis/kyphoscoliosis (40, 14.8%), revisions (20, 9.5%), and congenital (11, 5.4%). In the historic cohort kyphosis/kyphoscoliosis had the largest number of alerts (21, 9.3%), followed by syndromic (6, 4.2%), and then revisions (10, 3%), Table 2.

**Table 1:** Breakdown of patients by diagnosis

Diagnostic group	No. of patients (1985–2008)	No. of patients (2008–2024)	*p* valve
	(*n* = 3436)	*(n* = 3316)	
Idiopathic scoliosis	1618 (47.0%)	1835 (55.3%)	0.001
Neuromuscular scoliosis	626 (18.2%)	480 (14.5%)	0.001
Revision	329 (9.6%)	211 (6.4%)	0.001
Kyphosis/kyphoscoliosis	226 (6.6%	270 (8.1%)	0.0139
Congenital	234 (6.8%)	202 (6.1%)	0.23
Spondylolisthesis	154 (4.5%)	98 (3.0%)	0.001
Syndrome	143 (4.2%)	134 (4.0%)	0.802
Fracture/trauma	47 (1.4%)	47 (1.4%)	0.865
Pathologic	31 (0.9%)	32 (1.0%)	0.787
Degenerative	28 (0.8%)	7 (0.2%)	0.001

**Table 2:** Monitoring outcomes by diagnostic category

Diagnostic group	1985–2008			2008–2024		
	Total patients	TP (*n*, %)	FN (*n*, %)	Total patients	TP (*n*, %)	FN (*n*, %)
Kyphosis/kyphoscoliosis	226	21 (9.3%)	1 (0.4%)	270	40 (14.8%)	0
Syndrome	143	6 (4.2%)	0	134	7 (5.2%)	0
Revision	329	10 (3.0%)	1 (0.3%)	211	20 (9.5%)	1 (0.47%)
Neuromuscular scoliosis	626	16 (2.6%)	0	480	14 (2.9%)	0
Congenital	234	6 (2.6%)	1 (0.4%)	202	11 (5.4%)	0
Spondylolisthesis	154	3 (1.9%)	4 (2.6%)	98	0	0
Idiopathic scoliosis	1618	12 (0.7%)	0	1835	43 (2.3%)	4 (0.22%)
Fracture/trauma	47	0	0	47	0	0
Pathologic	31	0	0	32	0	0
Degenerative	28	0	0	7	0	0
Total	3436	74 (2.2%)	7 (0.2%)	3316	135 (4.1%)	5 (0.2%)

*TP* true positive, *FN* false negative

The alert modalities changed between the two cohorts. Most notably, in the modern era almost half the alerts were TCeMEP only (59, 43.7%), whereas there were zero in the historic alerts. The second most alerts were SSEP/DNEP (25, 18.5%) followed by DNEP only (24, 17.8%). For the earlier cohort SSEP only was the primary alert modality (16, 35%), followed closely by DNEP only (33.5%), and then SSEP/DNEP (20, 27%). Only 10.4% (14) of the alerts were SSEP only from modern period. Further details are provided in Table 3. The transition from DNEP to TCeMEP for motor tract monitoring occurred in the early phase of the modern cohort, with TCeMEP being the primary motor tract monitoring for all pediatric spine deformity cases since 2018.

**Table 3:** Alert modality

Alert modality	No. of patients 1985–2008	No. of patients 2008–2024
	(*n* = 74)	(*n* = 135)
SSEP only	26 (35%)	14 (10.4%)
DNEP only	25 (33.5%)	24 (17.8%)
SSEP/DNEP	20 (27%)	25 (18.5%)
SSEP/TCeMEP	1 (1.5%)	9 (6.7%)
SSEP/EMG	1 (1.5%)	0
SSEP/DSEP	1 (1.5%)	0
TCeMEP only	0	59 (43.7%)
TCeMEP/DNEP	0	4 (3.5%)

Of the 3316 patients in the modern cohort, 135 patients or 4.1% of this population had alert events during surgery. In the historic cohort of 3436, 74 patients, or 2.2%, had neuromonitoring alerts. Loss or significant degradation of monitoring data in cases of alerts were attributed to various causes. They are listed by frequency of occurrence, with reference to the historic study provided in Table 4. It should be noted that the degenerative cases included spondylolysis, and pathologic cases included tumors, bone cysts, cauda equina syndrome, infections from prior spine surgery, pathologic fracture, dysplastic scoliosis, and pathologic stenosis.

**Table 4:** Causes of alerts

Causes	No. of patients (1985–2008)	No. of patients (2008–2024)
	(*n* = 74)	(*n* = 135)
Correction/instrumentation	35 (47%)	84 (62.2%)
Osteotomy/decompression	Not reported	35 (25.9%)
Depth of anesthesia	Not reported	7 (5.2%)
Peripheral limb malposition/pressure	6 (8%)	6 (4.4%)
Hypotension	11 (15%)	3 (2.2%)
Wound or nerve root retraction	11 (15%)	0
Halo or femoral traction	7 (10%)	0
Hemostatic matrix in spinal canal	2 (3%)	0
Malignant hypothermia	1 (1%)	0
Partially occluded endotracheal tube	1 (1%)	0

Among the 135 alerts, 120 (88.9%) awoke with unchanged neurologic status while 15 (11.1%) awoke with changes in neurologic status; this excludes false negatives. Twelve of these 15 patients (8.9% of the alerts) had permanent neurologic deficits: nine were spinal cord deficits and three were nerve root in origin. The other three patients (2.1% of the alert group) awoke with transient postoperative neurologic deficits. Of these three transient deficits, one had a unilateral right upper extremity deficit, one had right lower extremity weakness, and one had monoparesis of the right upper extremity. The monitoring data for all three transient deficit patients remained within warning values throughout the procedure. Only one of these patients had intraoperative Stagnara wake-up test. Results were positive for this one patient. Transient deficits were resolved within 48 h for two of the three patients, while one patient the return time was not documented. Table 5 provides a breakdown of this group by details for the historic cohort and Table 6 provides a breakdown of this group by details for the modern cohort. When looking at permanent deficits from a perspective of diagnosis in the modern era, most occurred in kyphosis/kyphoscoliosis patients with eight deficits (1 FN, 7 TP), representing 17.5% of alerts for this group. The next most common was AIS, with six deficits (3 FN, 3 TP), representing a deficit rate of 7.0%.

**Table 5:** Permanent Deficit Patients (Historic Cohort: 1985 to 2008)

Diagnosis	Surgery	Etiology	Data	Intervention	Stagnara	Deficit
Neuromuscular scoliosis	PSF T2-ilium	Correction	LE DNEP and SSEP	Release correction	No	Paraplegia
Adolescent idiopathic scoliosis	PSF T3–T11	Screw placement	LE DNEP and SSEP	Increased MAP, instrumentation removed	Yes, positive for LLE	Altered sensation
Scheuermann’s kyphosis	ASF and PSF	Correction	DNEP and SSEP	Instrumentation removed	Yes, positive bilaterally	Paraparesis
Congenital kyphoscoliosis	PSF T–T12 with T7 VCR	Correction	DNEP and LE SSEPs	Traction decreased, instrumentation removed	Yes, positive	Paraplegia
Grade IV spondylolisthesis	PSF L4–S1, spondylolisthesis reduction	Reduction	L SSEP and L4–L5 dermatome	Distraction reduced on L side	Yes L > R	L foot drop

*DNEP* descending neurogenic-evoked potentials, *SSEP* somatosensory-evoked potential, *PSF* posterior spinal fusion, *ASF* anterior spinal fusion, *VCR* vertebral column resection, *LE* lower extremity, *MAP* mean arterial pressure, *L* left, *R* right

**Table 6:** Permanent deficit patients (Modern cohort: 2008–2024)

Diagnosis	Surgery	Etiology	Data	Intervention	Stagnara	Deficit
Adolescent idiopathic scoliosis	PSF T2–L4	Correction	SSEP DNEP	Increased BP, traction decreased, procedure aborted	Yes, positive LLE	Paraparesis
Adolescent idiopathic scoliosis	PSF T2–L4	Osteotomy/decompression	SSEP TCeMEP	Decreased anesthetic, procedure aborted	Yes, positive LLE	Paraparesis
Adolescent idiopathic scoliosis	PSF T2–L4	Correction	DNEP	Removed instrumentation, procedure aborted	Yes, positive BLE	Paraplegia
Congenital kyphosis	PSF T3–12; VCR T6–8	Correction	DNEP	None	Yes, positive BLE	Paraparesis
Congenital Scoliosis	PSF T2–L3; osteotomies T5–8; VCR T5; decompression T4–6	Correction	TCeMEP DNEP	Released Correction	No	Paraplegia
Kyphosis	PSF T2–12 with VCR T7	Osteotomy/decompression	SSEP DNEP	Increased BP	Yes, positive BLE	Paraplegia
Kyphoscoliosis	PSF C7-L1; VCR T5–6	Osteotomy/decompression	SSEP	Further decompression	Yes, positive BLE	Paraplegia
Kyphoscoliosis	PSF occiput—T5	Correction	DNEP	None	Yes, positive BLE	Paraparesis
Kyphoscoliosis	PSF T1–L4; VCR T10	Osteotomy/ decompression	SSEP DNEP	Further decompression	Yes, negative	Altered sensation both feet
Scheuermann’s kyphosis	PSF C6–L3; Vertebrectomy T3	Peripheral limb malposition	SSEP	None	No	Altered sensation LUE
Scheuermann’s kyphosis	PSF T3–L3; osteotomies T7–11	Correction	SSEP DNEP	Reposition Pt, release correction	Yes, negative	Altered sensation RUE
Syndromic scoliosis	PSF T2–L3; osteotomies T6–9	Screw placement	DNEP	Removed instrumentation, procedure aborted	Yes, positive BLE	Paraparesis

*DNEP* descending neurogenic-evoked potentials, *SSEP* somatosensory-evoked potential, *TCeMEP* transcranial electrical motor-evoked potentials, *PSF* posterior spinal fusion, *VCR* vertebral column resection, *LE* lower extremity, *UE* upper extremity, *MAP* mean arterial pressure, *L* left, *R* right, *BLE* bilateral lower extremity, *Pt*, patient, *BP* blood pressure

Four patients (0.12% of all the patients) had events in which no intervention was required, and no neurologic deficit occurred postoperatively. These cases were classified as false positives. Five patients (0.15%) had false-negative monitoring outcomes. These patients awoke with neurologic deficits undetected by neuromonitoring. One of the five deficits was a transient motor nerve root deficit that was resolved by 6-week post-op. Four of the five patients had permanent deficits: one with paraplegia and three with a combination of sensory loss and weakness. Granular data of these false-negative cases can be found in Table 7. In the historic cohort there were seven false positives (0.2%). There were a similar number of false negatives (7, 0.2%); however, only one had a permanent deficit- loss of left quadriceps motor function.

**Table 7:** False-negative patients (Modern cohort: 2008–2024)

Diagnosis	Surgery	Data	Deficit
Adolescent idiopathic scoliosis	PSF T3-L2	SSEP	Permanent R hemiparesis
		DNEP	
Kyphoscoliosis	PSF T1–L4, VCR T9–T11	SSEP	Permanent paraplegia
		DNEP	
Adolescent idiopathic scoliosis	PSF T9–L4	SSEP	Permanent RLE quadriceps weakness and altered sensation
		DNEP	
Adolescent idiopathic scoliosis	PSF T10–L4	SSEP	Permanent RLE weakness and altered sensation
		DNEP	
Adolescent idiopathic scoliosis	PSF T2–T12	SSEP	Transient L foot drop
		TCeMEP	

*DNEP* descending neurogenic-evoked potentials; *SSEP* somatosensory-evoked potential, *TCeMEP* transcranial electrical motor-evoked potentials, *VCR* vertebral column resection, *PSF* posterior spinal fusion, *RLE* right lower extremity, *L* left, *R* right

A total of 16 patients (0.48%) had permanent neurologic deficits. Twelve of the permanent deficits that occurred were alert events, and four were false-negative events. The ratio of intraoperative alert events to total patients in the modern cohort was 1 in 24 (4.1%), while the observed permanent neurologic deficit rate was 0.48% (1 in 207). In the historical cohort, the permanent deficit rate was 0.17% (1 in 573), with only one false-negative event. The ratio of intraoperative alert events to total patients in the historical cohort was 1 in 46 (2.2%).

Although not fully reported in the original Thuet study, the rates and types of vertebral osteotomies and use of pre-operative halo-gravity traction were evaluated to better characterize potential differences in case complexity between the two eras. Of note, there were no alerts due to osteotomy/decompression in the historic cohort; vertebral column resection (VCR) was reported only among patients with permanent neurologic deficits, with a single VCR case resulting in paraplegia. In the modern cohort, 40 alerts were in cases with VCR (29.6% of alerts—three were three vertebral levels, six were two levels, and the rest were one level. Six of these patients ultimately sustained permanent neurologic deficits (15% of alerts with VCR, 37.5% of permanent deficits). Other common osteotomies in the modern cohort alerts include 56 posterior column (mean number of levels 6.9 ± 3), one pedicle subtraction (one level), and one hemi-vertebrectomy (three level). In the modern cohort, 43 patients with alerts underwent treatment with traction prior to fusion surgery, five of these had permanent deficits (31.3% of permanent deficits). Pre-operative traction was not reported in the historic cohort.

In terms of test parameters, there were 135 true positives in the modern cohort, and 74 in the historic. There were four false positives in the modern cohort and seven in the historic. Additionally, there were 3172 true negatives in the modern cohort and 3348 in the historic. Lastly, five false negatives in the modern cohort and seven in the historic. These differences translated into improved test performance in the modern era, with higher sensitivity (96.43% vs 91.36%), specificity (99.87% vs 99.79%), positive predictive value (97.12% vs 91.34%), and negative predictive value (99.84% vs 99.79%) compared with the historic cohort.

## Discussion

Intraoperative monitoring is used as a tool to reduce potential neurologic deficit in a variety of surgical procedures [4, 8] The initial 2010 retrospective study by Thuet et al. (1985–2008) established the benefit of multimodality IONM in identifying and reducing permanent neurologic deficits across a broad spectrum of pediatric spinal deformities between 1985 and 2008. This current study aimed to extend that analysis, examining whether the benefits and predictive performance of IONM have remained consistent, improved, or plateaued in the subsequent period from 2008 to 2024, given advancements in both surgical techniques and monitoring modalities. Our modern cohort (3316 pediatric spinal surgeries) confirms IONM’ s vital role in neurologic protection but also demonstrated notable shifts in IONM modalities utilized, alert frequency and outcomes.

Thuet et al. reported 74 (2.2%) positive events, with six permanent neurologic deficits (0.17%). Our cohort shows a higher rate of alert events (135 patients, 4.1%). Despite increased detection, permanent neurologic deficits in our study were 16 patients (0.48%). While the absolute number and percentage of permanent deficits increased in the modern era, this shift likely reflects an increase in baseline surgical risk. The modern cohort consists of more complex cases, increased medical comorbidities, evolution in surgical technique or goals of correction. The transition from universal instrumentation and all hook-constructs to hybrid and ultimately all-pedicle-screw constructs has dramatically changed how correction is achieved and the degree of correction possible. Concurrently, there has been a marked decline in anterior approaches, with the rise in the use of three-column osteotomies, femoral/halo traction, and vertebral column resections (VCRs), particularly in complex or rigid deformities. These trends remained evident in the results of this study with large number and complexity of osteotomies as well as patients undergoing treatment with traction preoperatively. All these procedures inherently carry higher neurologic risks.

As noted in the original paper, IONM is not without its weaknesses. There were five patients in our study who had events that went unnoticed by IONM. Thuet et al. reported seven (0.2%) false-negative events, with one permanent deficit (left quadriceps motor function loss). Our study found five (0.15%) false-negative events, but four resulted in permanent deficits (one paraplegia, three with sensory loss and weakness). While the overall false-negative rate decreased, the proportion leading to permanent deficit significantly increased (80 vs. 14% in Thuet). This highlights IONM’s potential insufficiency, especially for subtle or rapid deficits, or nerve root level compromises, as Thuet et al. also suggested. The prior study noted 6 of 7 false negatives, including the only permanent neurologic deficit, were nerve root in origin; our data with 3 of 4 false negatives involving permanent sensory loss and weakness further supports this.

Correction or instrumentation was the leading cause of monitoring data degradation in both studies (47% in Thuet et al. vs 62.2% in our study). This consistency confirms mechanical aspects of deformity correction as the primary neurologic risk. Our study also shows a significant percentage attributed to osteotomy/decompression (25.9%) suggesting, as aforementioned, destabilizing osteotomies and decompressions may contribute to intraoperative events. This is consistent with existing literature which suggests more rigid deformities with increased correction via osteotomy, are at higher risk of neurologic complications postoperatively [9]. Hypotension, a significant associated factor for IONM alerts in the original study (15%), was less frequent in our cohort (2.2%), likely due to improved anesthetic and hemodynamic management, and the recognition of hypotension on spinal cord perfusion. A 2007 study by Schwartz et al. reported hypotension as the cause of 9 of 38 alerts (~ 24%) in scoliosis surgery.^10^ This study was conducted during an era when deliberate hypotension was more commonly employed to reduce surgical blood loss [10]. Since then, anesthesia practice has shifted toward maintaining higher MAP targets (generally > 65–70 mmHg) as well as protocols addressing blood pressure first upon any signal change [11, 12]. This would directly reduce hypotension-triggered alerts.

Both studies show certain diagnostic groups have higher event risks. Thuet et al. found kyphosis/kyphoscoliosis (9.3%), syndromic (4.2%), and revision (3.0%) cases had the highest percentages of alerts. Our data similarly shows high alert rates for kyphosis/kyphoscoliosis (14.8%), revision (9.5%), and congenital (5.4%) scoliosis/kyphosis cases. Furthermore, 17.5% of alerts for the kyphosis/kyphoscoliosis group resulted in permanent deficit. The significant increase in alert events for kyphosis/kyphoscoliosis and revisions in our cohort suggests these deformities continue to pose substantial neurologic risk and/or that monitoring is now more sensitive [13]. Furthermore, this supports literature which has demonstrated that kyphosis/kyphoscoliosis has an impact on cord morphology, and thus higher risk of neurologic risk intraoperatively [14, 15]. The original study noted low risk in fracture/trauma, pathologic, and degenerative groups due to small sample sizes; our study, with slightly larger numbers, still shows no alert or false-negative events, reinforcing their lower-risk nature, though caution remains.

One of the biggest shifts between the two time periods was the modalities used for IONM. Thuet et al. identified SSEP (35%) and DNEP (33.5%) as primary alert modalities, with combined SSEP/DNEP at 27%. TCeMEP was a sole warning criterion less frequently (1.5%). Our data show a significant shift: TCeMEP alone accounted for 43.7% of alert events, becoming the most frequent single alert modality. Combined SSEP/DNEP (18.5%), DNEP only (17.8%), and SSEP only (10.4%) also contributed. This shift indicates increased reliance on and improved sensitivity of TCeMEP for motor pathway compromise, aligning with its broader adoption since 2008. TCeMEPs were not as widely used prior to this time due to the need for special equipment and its sensitivity to commonly used anesthetics [13, 16]. Both of these have been mitigated with the advances in IONM technology as well as anesthetic technique [13]. Furthermore, multiple studies have demonstrated TCeMEPs increased sensitivity [10, 15, 17]. There was a large shift away from the use of DNEPs after 2000 once a study by Toleikis et.al demonstrated their largely sensory components [18]. DNEPs also do not detect ischemia of the area supplied by the anterior spinal artery which can lead to complete motor paralysis below the level of the lesion (anterior cord syndrome) [18]. While DNEP remains in a more limited role often used in combination with other modalities, TCeMEPs rise is a key difference between the eras. Interestingly, in the modern era, only two patients monitored with TCeMEPs developed permanent neurologic deficits, and only one case represented a false-negative result.

Buckwalter et al. demonstrated that combined TCeMEP and SSEP monitoring not only detects true intraoperative compromise but also allows corrective actions that largely prevent postoperative deficits, underscoring the utility of TCeMEP in modern deformity surgery [19]. However, while advances in anesthetic management have improved hemodynamic stability and overall monitoring reliability, it is important to note that TCeMEPs still remain more sensitive to anesthetic depth and agents than other modalities [15]. Some alerts therefore may represent transient or anesthetic related changes rather than true neurologic deficit. However, distinguishing false positives from true alerts intraoperatively remains a challenge, due to longer more complex surgeries and “anesthetic fade”—a phenomena in which signal degradation occurs with longer anesthetic time [20]. The increased use of TCeMEPs in the recent era, with its sensitivity to anesthesia, has contributed to the increasing frequency of managing “anesthetic fade.” This ultimately necessitates a cautious, intervention-based approach to any IONM change. Implementing flowcharts and checklists have been successful in reducing numbers of TP and FP cases [12, 21, 22], and should be continually be refined in order to further mitigate neurologic risk in pediatric spine deformity surgery.

There are some limitations to note in this study. Both retrospective and single-institution analyses limit generalizability. Direct comparisons are challenging due to different surgeons between the eras, evolving surgical techniques, anesthetic protocols, and monitoring equipment over decades, thus this study is limited to descriptive outcomes overtime. Different institutions, and even providers within these institutions, have varied protocols when it comes to IONM and anesthesia. Importantly, the absence of an untreated comparator cohort limits definitive conclusions regarding the natural history of intraoperative neuromonitoring (IONM) alerts and the true magnitude of neurologic risk reduction attributable to monitoring. While the frequency of alert events substantially exceeded the observed rate of permanent neurologic deficits, false-negative monitoring remains an inherent limitation, and the true incidence of neurologic injury may therefore be under- or overestimated. Consequently, causality cannot be definitively established, and these findings should be interpreted cautiously. Lastly, a notable limitation of this study is the inability to perform adjusted multivariate analysis across diagnostic subgroups. Because permanent neurologic deficits remain a rare event (0.48%), the resulting small subgroup sizes preclude definitive causal modeling between specific diagnoses and monitoring outcomes.

This descriptive cohort study affirms the enduring value of multimodal IONM in pediatric spinal deformity surgery over nearly four decades. While the contemporary era demonstrated a higher proportion of false-negative events resulting in permanent neurologic deficits, this trend likely reflects the increased complexity of modern cases—including greater use of vertebral column resections and traction—rather than a decline in monitoring efficacy. Descriptively, calculated monitoring sensitivity and overall test performance appeared similar or slightly improved in the contemporary cohort; however, these findings should be interpreted cautiously given substantial differences in case complexity and clinical practice between eras. Another notable shift in the eras includes the change from DNEP to TCeMEP. Continuous vigilance, refinement of monitoring techniques/protocols, especially for nerve root integrity, and understanding evolving risk profiles from complex surgeries are paramount to minimizing iatrogenic neurologic complications in this vulnerable patient population.

## Supplementary Information

Below is the link to the electronic supplementary material.

Supplementary file1 (DOCX 17 KB)

## Data Availability

The datasets generated during and/or analysed during the current study are available from the corresponding author on reasonable request.
