# Supplementary material for: Evolution of multimodal intraoperative neuromonitoring in pediatric spinal deformity surgery over 39 years: are we better?
Source: Spine Deform. 2026 Jun 11;14(6):2083–91. doi: 10.1007/s43390-026-01453-4 (PMC13690883; doi:10.1007/s43390-026-01453-4)
Supplement: Supplementary file 1 [file 43390_2026_1453_MOESM1_ESM.docx]

**Appendix**:

For the lower extremity SSEPs, stimulation was of the posterior tibial nerve at the medial malleolus or sciatic nerve at the popliteal fossa. Upper extremity SSEPs were elicited via stimulation of the ulnar nerve at the cubital tunnel and/or the median nerve at the wrist crease. Stimulation was done via pre-gelled patch electrodes. Subdermal needle electrodes (12 mm) were used for all SSEP recording sites. According to the International Federation 10–20 electrode system, far-field responses were recorded across the somatosensory cortex at C1, C2, CZ, C3, and C4 across the somatosensory cortex. Additionally, a subcortical recording was obtained at the occiput.

**For** upper extremity responses **TCeMEPs** were recorded bilaterally from the abductor digiti minimi and abductor pollicis brevis. Lower extremity recording sites were the adductor longus, vastus medialis, tibialis anterior, medial gastrocnemius, extensor hallucis longus, and abductor hallucis muscles. Data were elicited via constant-voltage current delivered through 2 subdermal needle/corkscrew electrodes placed subcutaneously over the motor cortex at C3–C4 with the 10–20 System. Stimulation was delivered as a train of 7–9 anodal pulses, with each pulse lasting 50–75 µs and spaced 1–5 ms apart. The stimulus intensity was gradually increased until a threshold-level response was elicited. The intensity of stimulation ranged between 75 and 500V. A short-acting muscle relaxant at induction via total intravenous anesthetic was administered, when TCeMEP monitoring was used, to obtain pre-incision baseline data. A second dose of muscle relaxants was given for surgical dissection and then the patient was allowed to return to 4 strong twitches for the remainder of the surgical procedure. Each recording site’s baseline was defined by the lowest voltage that reliably produced a compound muscle action potential. Following surgical exposure, once it was confirmed the patient had 4 strong twitches with a standard train-of-four peripheral stimulator, the baseline values were determined. Any threshold increase of ≥100 V from baseline was designated as a warning criterion.

**Descending neurogenic-evoked potentials (DNEPs)** were used in surgeries based on surgeon preference. Stimulation was performed by 1 of 3 methods:

1. **Percutaneous Stimulation (PERC):** Achieved through the insertion of two 27-gauge needle electrodes (50–75 mm length) over neighboring cervical laminae using a percutaneous approach.
2. **Epidural Stimulation (EPI-DNEP):** Conducted with a flexible spinal catheter containing a pair of 1.3 mm platinum electrodes spaced 1.5 cm apart (Ad-Tech Medical Instrument Corp, Racine, WI). The catheter was advanced cranially through the ligamentum flavum at the cephalad margin of the instrumentation to position the electrodes above all operative spinal levels.
3. **Spinous Process Stimulation (SP-DNEP):** Performed by placing two 24-gauge, 12 mm needle electrodes directly into the spinous processes of sequential vertebrae at the upper extent of the surgical incision.

Constant current or voltage was applied in an ascending manner to obtain a consistent, repeatable DNEP response. All DNEP data were recorded bilaterally from the sciatic nerves at the popliteal fossa.

Pre-incision recordings for all monitored modalities were obtained following patient positioning on the operative table and prior to skin incision. These initial measurements confirmed the presence of reliable evoked potential responses. Baseline values were then established after surgical exposure was complete but before any maneuvers that could jeopardize neural structures began. Data susceptible to intraoperative changes were continuously elicited and recorded throughout the procedure. Monitoring was maintained during wound closure and continued for at least 30 minutes after the correction was completed.

Warning criteria for SSEP were defined as a reduction in amplitude of 60%, a latency increase of 10%, or both, relative to baseline measurements. For DNEP, warning thresholds were set at an 80% decrease in amplitude, a 10% increase in latency, or both. Upon meeting these criteria, technical issues such as equipment malfunction were first ruled out. Subsequently, anesthetic levels, blood loss, and mean arterial pressure were assessed. The surgeon was then notified of the neurophysiological changes along with the patient’s current physiological status.

When needed, a **Stagnara wake-up test** was ordered to confirm motor status intraoperatively. A positive wake-up test was defined as a patient who demonstrated no movement from one or both lower extremities but had normal upper extremity function. A wake-up test was considered negative when a patient demonstrated normal motor function of all four extremities on command.

**Spontaneous electromyography (EMG) was used in cases where individual nerve roots were at risk of deficit- specifically the cervical and lumbar regions. Therefore, it is used in all surgeries with fusion and decompression to monitor specific nerve root function, providing real-time analysis during surgery. Triggered EMG was used to assess pedicle screw placement in the thoracolumbar spine, with recording muscles selected based on at-risk or operative spinal levels.**

While each anesthesia provider has slightly varied protocols, most include the following:

- Propofol infusion
- Methadone, Fentanyl, Remifentanil, and other narcotics via bolus
- Vecuronium or rocuronium administered as repeated bolus injections to maintain muscle relaxation
